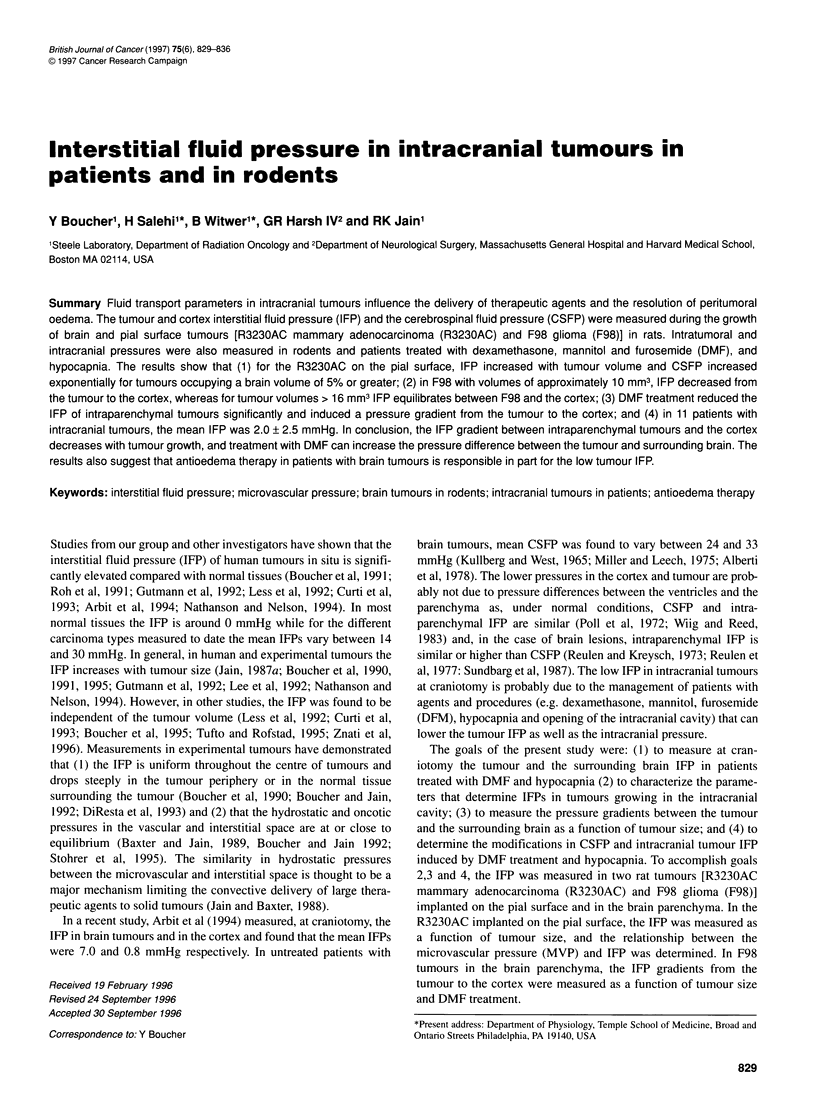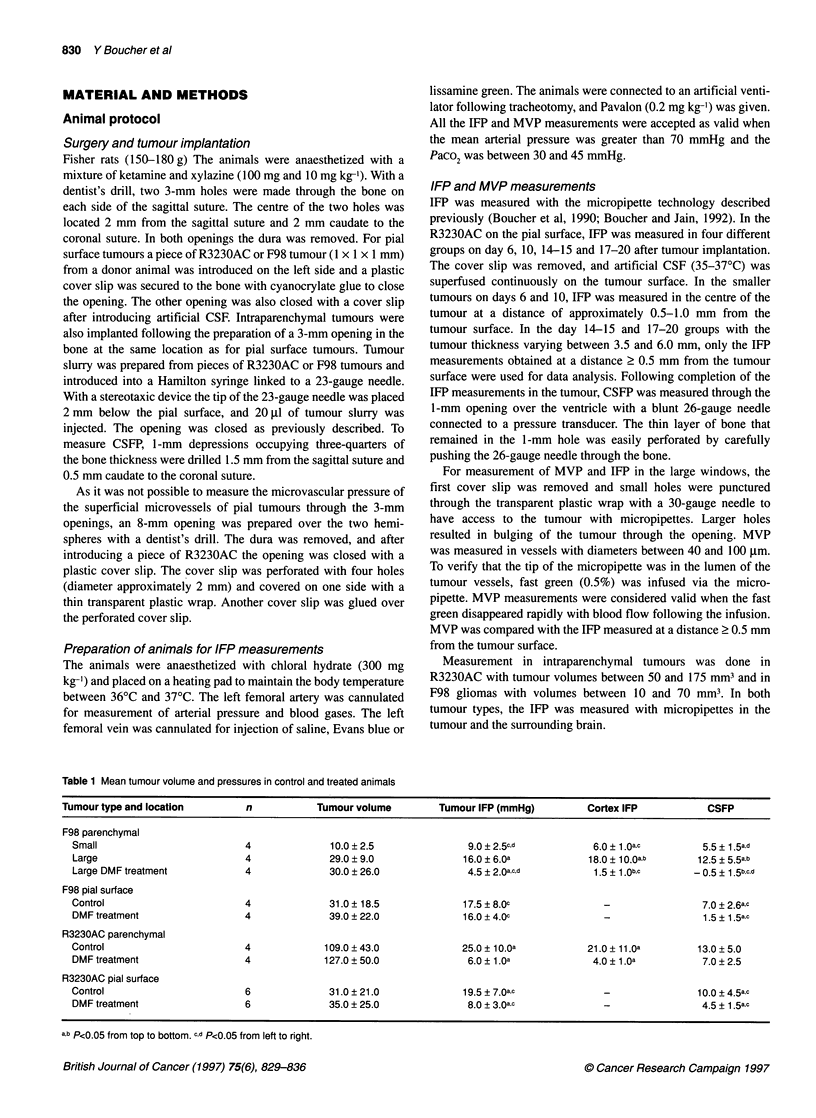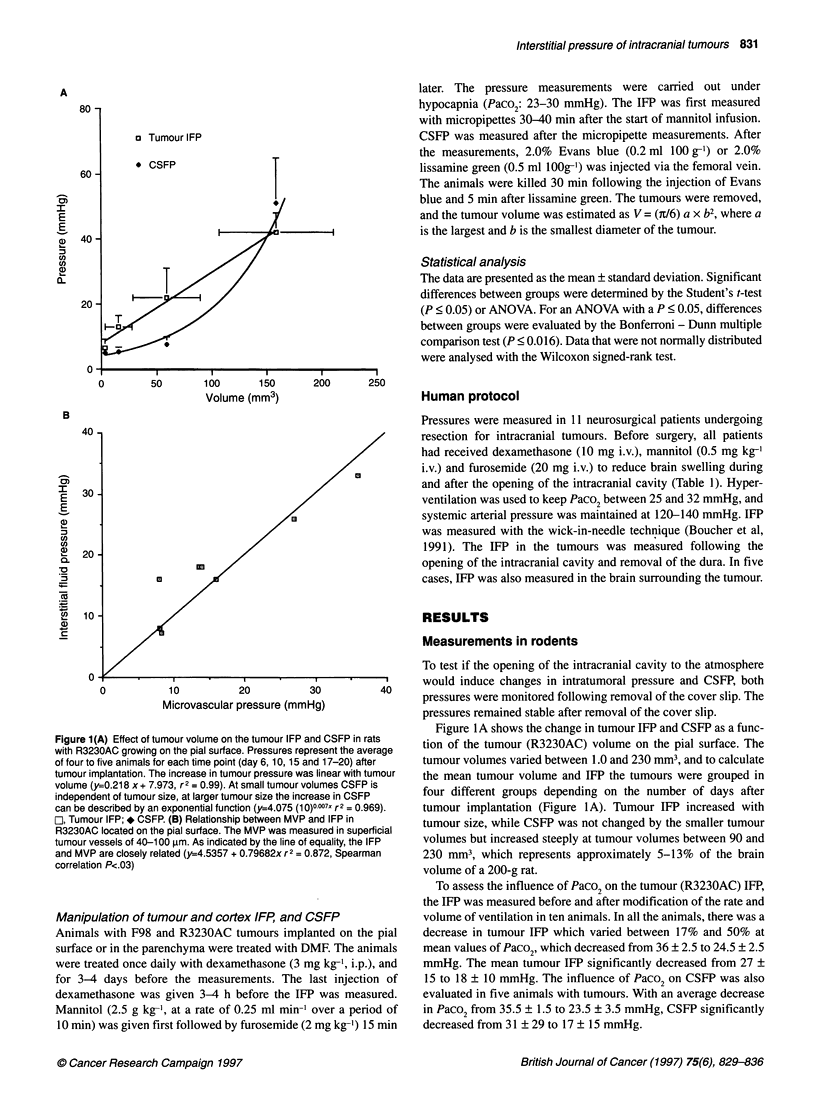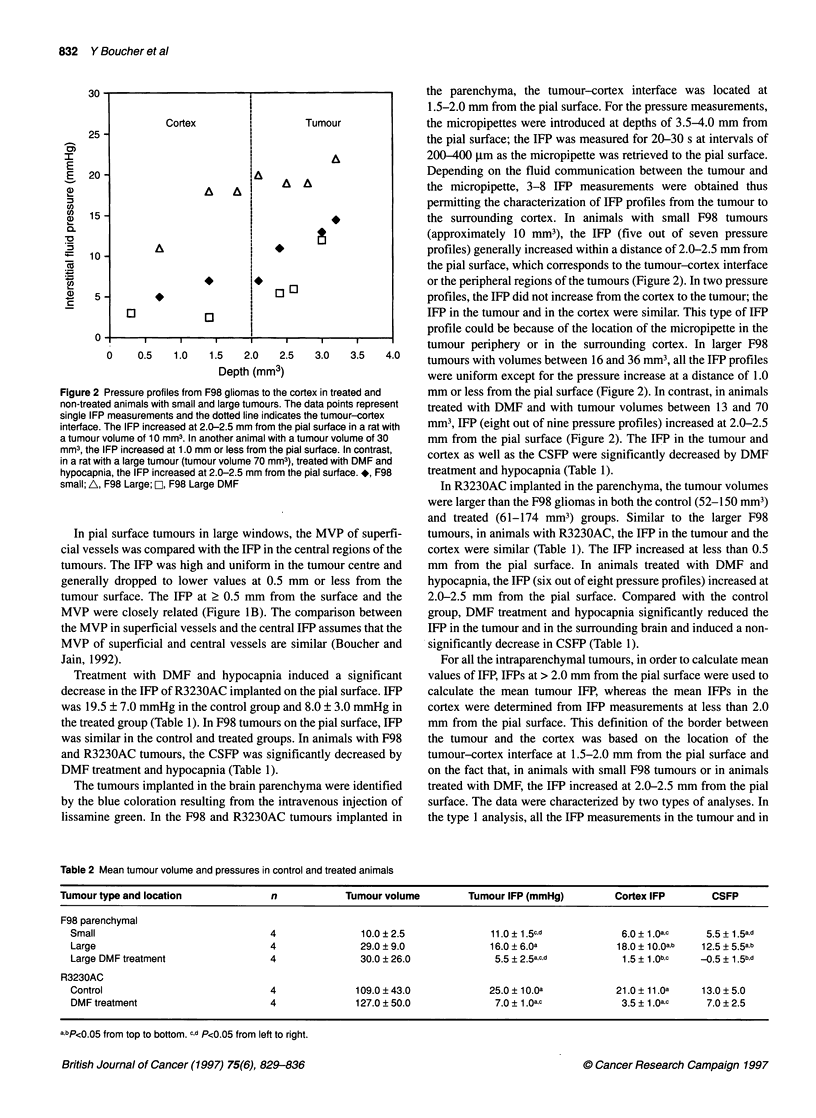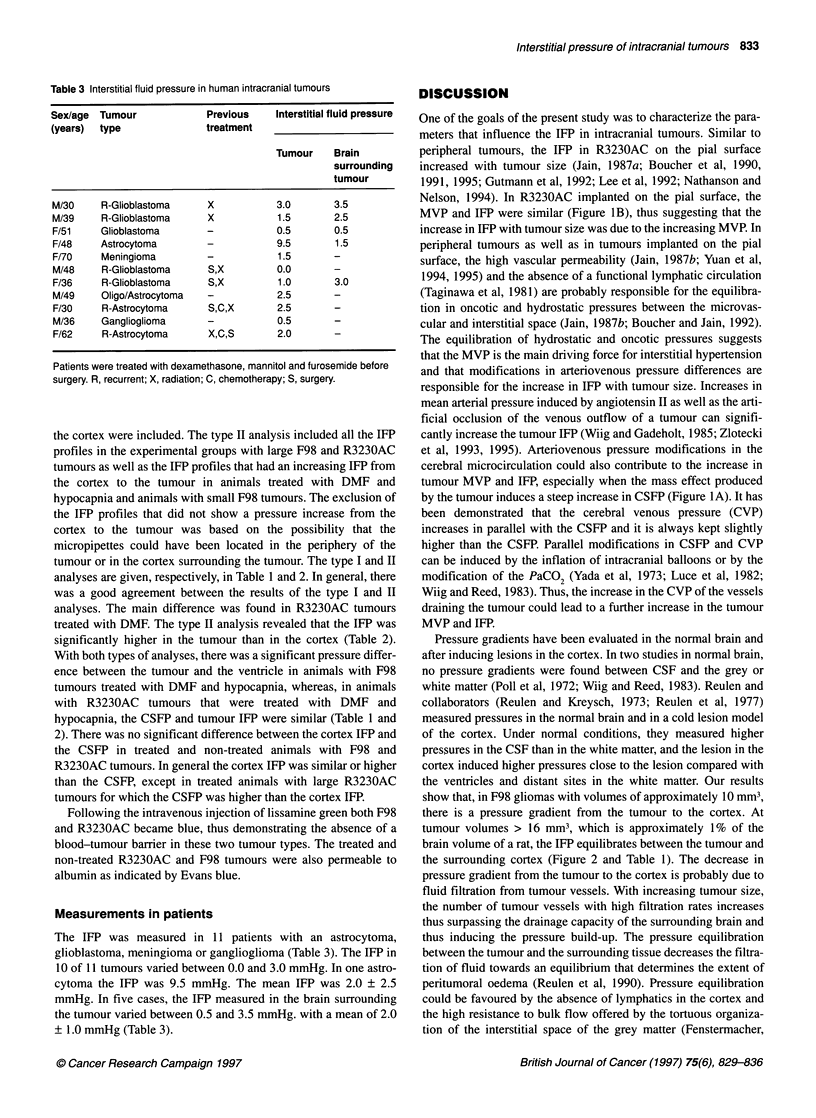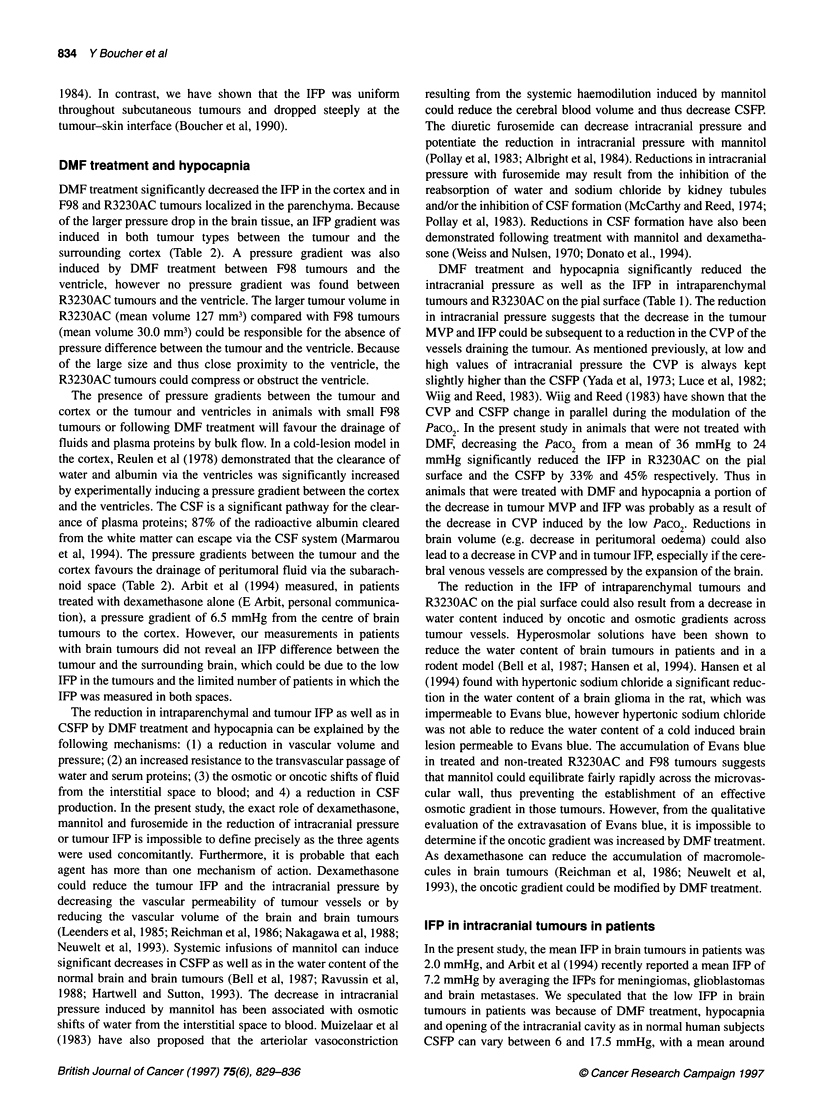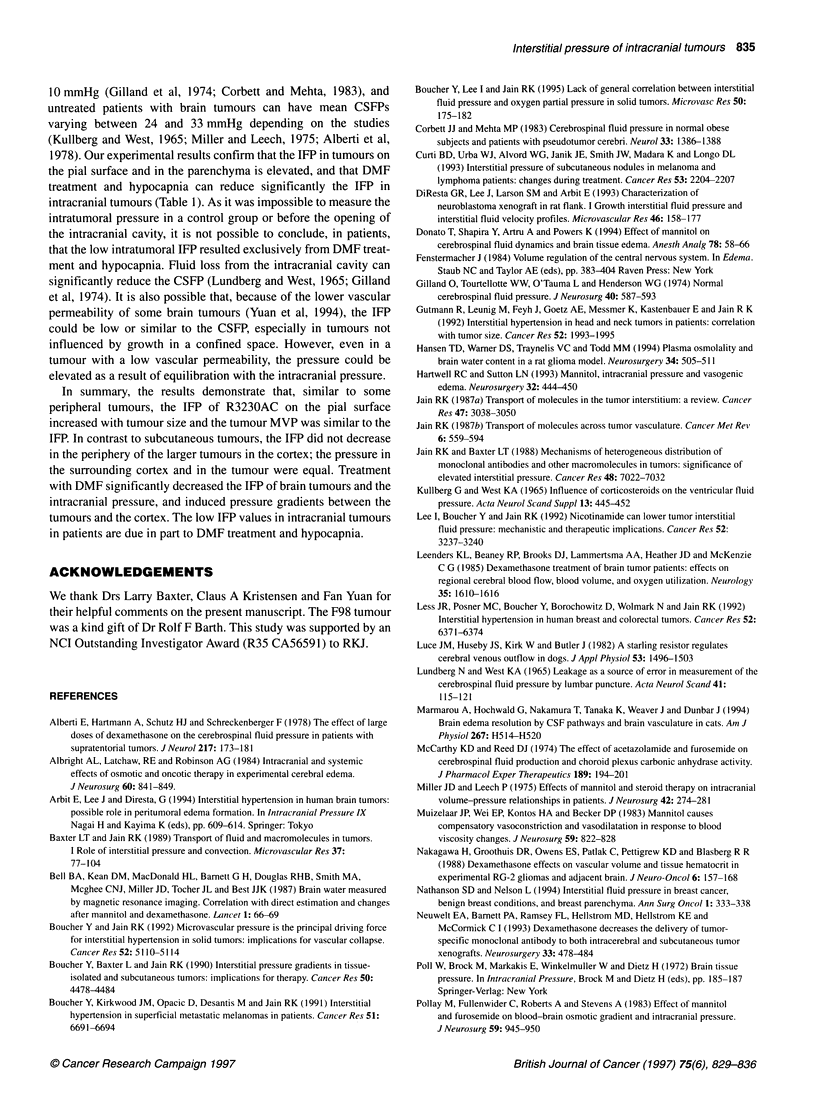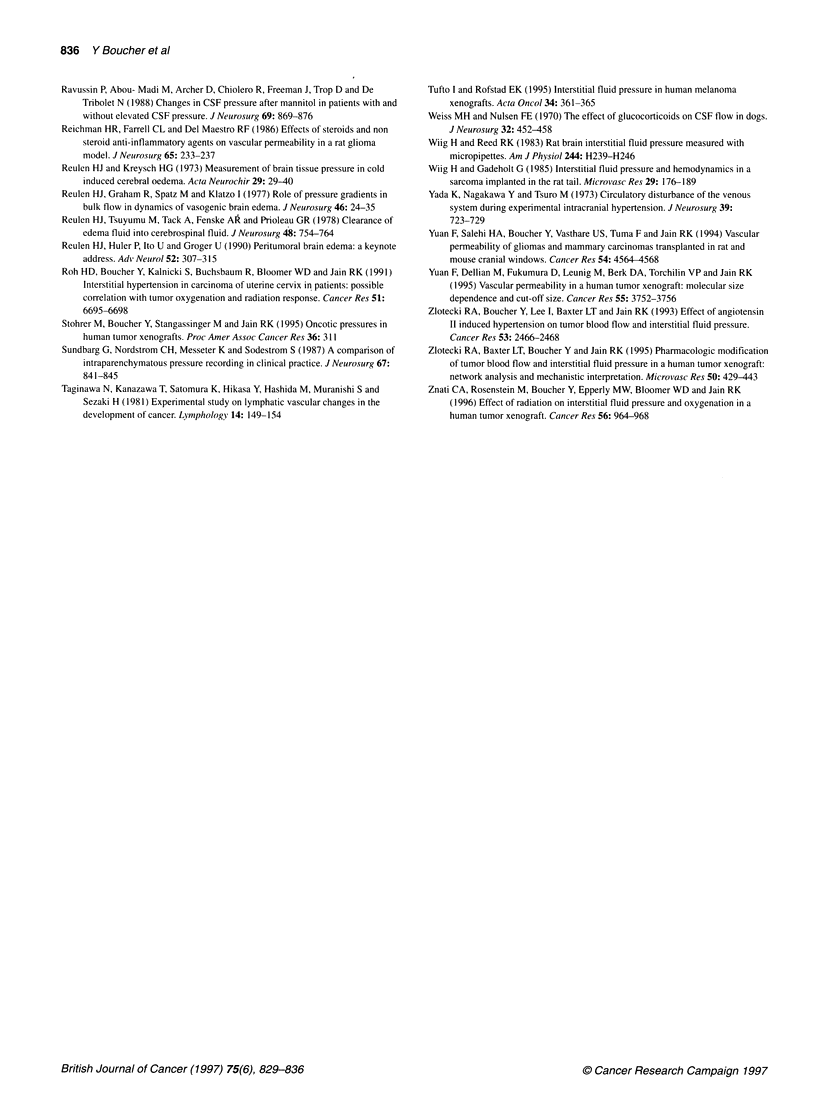# Interstitial fluid pressure in intracranial tumours in patients and in rodents.

**DOI:** 10.1038/bjc.1997.148

**Published:** 1997

**Authors:** Y. Boucher, H. Salehi, B. Witwer, G. R. Harsh, R. K. Jain

**Affiliations:** Department of Radiation Oncology, Massachusetts General Hospital and Harvard Medical School, Boston 02114, USA.

## Abstract

Fluid transport parameters in intracranial tumours influence the delivery of therapeutic agents and the resolution of peritumoral oedema. The tumour and cortex interstitial fluid pressure (IFP) and the cerebrospinal fluid pressure (CSFP) were measured during the growth of brain and pial surface tumours [R3230AC mammary adenocarcinoma (R3230AC) and F98 glioma (F98)] in rats. Intratumoral and intracranial pressures were also measured in rodents and patients treated with dexamethasone, mannitol and furosemide (DMF), and hypocapnia. The results show that (1) for the R3230AC on the pial surface, IFP increased with tumour volume and CSFP increased exponentially for tumours occupying a brain volume of 5% or greater; (2) in F98 with volumes of approximately 10 mm3, IFP decreased from the tumour to the cortex, whereas for tumour volumes > 16 mm3 IFP equilibrates between F98 and the cortex; (3) DMF treatment reduced the IFP of intraparenchymal tumours significantly and induced a pressure gradient from the tumour to the cortex; and (4) in 11 patients with intracranial tumours, the mean IFP was 2.0 +/- 2.5 mmHg. In conclusion, the IFP gradient between intraparenchymal tumours and the cortex decreases with tumour growth, and treatment with DMF can increase the pressure difference between the tumour and surrounding brain. The results also suggest that antioedema therapy in patients with brain tumours is responsible in part for the low tumour IFP.


British Journal of Cancer (1997) 75(6), 829–836
© 1997 Cancer Research Campaign

### Interstitial fluid pressure in intracranial tumours in patients and in rodents

**Y Boucher[1], H Salehi[1]*, B Witwer[1]*, GR Harsh IV[2] and RK Jain[1]**

[1]Steele Laboratory, Department of Radiation Oncology and [2]Department of Neurological Surgery, Massachusetts General Hospital and Harvard Medical School, Boston MA 02114, USA

**Summary** Fluid transport parameters in intracranial tumours influence the delivery of therapeutic agents and the resolution of peritumoral oedema. The tumour and cortex interstitial fluid pressure (IFP) and the cerebrospinal fluid pressure (CSFP) were measured during the growth of brain and pial surface tumours [R3230AC mammary adenocarcinoma (R3230AC) and F98 glioma (F98)] in rats. Intratumoral and intracranial pressures were also measured in rodents and patients treated with dexamethasone, mannitol and furosemide (DMF), and hypocapnia. The results show that (1) for the R3230AC on the pial surface, IFP increased with tumour volume and CSFP increased exponentially for tumours occupying a brain volume of 5% or greater; (2) in F98 with volumes of approximately 10 mm$^3$, IFP decreased from the tumour to the cortex, whereas for tumour volumes > 16 mm$^3$ IFP equilibrates between F98 and the cortex; (3) DMF treatment reduced the IFP of intraparenchymal tumours significantly and induced a pressure gradient from the tumour to the cortex; and (4) in 11 patients with intracranial tumours, the mean IFP was 2.0 ± 2.5 mmHg. In conclusion, the IFP gradient between intraparenchymal tumours and the cortex decreases with tumour growth, and treatment with DMF can increase the pressure difference between the tumour and surrounding brain. The results also suggest that antioedema therapy in patients with brain tumours is responsible in part for the low tumour IFP.

**Keywords:** interstitial fluid pressure; microvascular pressure; brain tumours in rodents; intracranial tumours in patients; antioedema therapy

Studies from our group and other investigators have shown that the interstitial fluid pressure (IFP) of human tumours in situ is significantly elevated compared with normal tissues (Boucher et al, 1991; Roh et al, 1991; Gutmann et al, 1992; Less et al, 1992; Curti et al, 1993; Arbit et al, 1994; Nathanson and Nelson, 1994). In most normal tissues the IFP is around 0 mmHg while for the different carcinoma types measured to date the mean IFPs vary between 14 and 30 mmHg. In general, in human and experimental tumours the IFP increases with tumour size (Jain, 1987*a*; Boucher et al, 1990, 1991, 1995; Gutmann et al, 1992; Lee et al, 1992; Nathanson and Nelson, 1994). However, in other studies, the IFP was found to be independent of the tumour volume (Less et al, 1992; Curti et al, 1993; Boucher et al, 1995; Tufto and Rofstad, 1995; Znati et al, 1996). Measurements in experimental tumours have demonstrated that (1) the IFP is uniform throughout the centre of tumours and drops steeply in the tumour periphery or in the normal tissue surrounding the tumour (Boucher et al, 1990; Boucher and Jain, 1992; DiResta et al, 1993) and (2) that the hydrostatic and oncotic pressures in the vascular and interstitial space are at or close to equilibrium (Baxter and Jain, 1989, Boucher and Jain 1992; Stohrer et al, 1995). The similarity in hydrostatic pressures between the microvascular and interstitial space is thought to be a major mechanism limiting the convective delivery of large therapeutic agents to solid tumours (Jain and Baxter, 1988).

In a recent study, Arbit et al (1994) measured, at craniotomy, the IFP in brain tumours and in the cortex and found that the mean IFPs were 7.0 and 0.8 mmHg respectively. In untreated patients with brain tumours, mean CSFP was found to vary between 24 and 33 mmHg (Kullberg and West, 1965; Miller and Leech, 1975; Alberti et al, 1978). The lower pressures in the cortex and tumour are probably not due to pressure differences between the ventricles and the parenchyma as, under normal conditions, CSFP and intraparenchymal IFP are similar (Poll et al, 1972; Wiig and Reed, 1983) and, in the case of brain lesions, intraparenchymal IFP is similar or higher than CSFP (Reulen and Kreysch, 1973; Reulen et al, 1977: Sundbarg et al, 1987). The low IFP in intracranial tumours at craniotomy is probably due to the management of patients with agents and procedures (e.g. dexamethasone, mannitol, furosemide (DFM), hypocapnia and opening of the intracranial cavity) that can lower the tumour IFP as well as the intracranial pressure.

The goals of the present study were: (1) to measure at craniotomy the tumour and the surrounding brain IFP in patients treated with DMF and hypocapnia (2) to characterize the parameters that determine IFPs in tumours growing in the intracranial cavity; (3) to measure the pressure gradients between the tumour and the surrounding brain as a function of tumour size; and (4) to determine the modifications in CSFP and intracranial tumour IFP induced by DMF treatment and hypocapnia. To accomplish goals 2,3 and 4, the IFP was measured in two rat tumours [R3230AC mammary adenocarcinoma (R3230AC) and F98 glioma (F98)] implanted on the pial surface and in the brain parenchyma. In the R3230AC implanted on the pial surface, the IFP was measured as a function of tumour size, and the relationship between the microvascular pressure (MVP) and IFP was determined. In F98 tumours in the brain parenchyma, the IFP gradients from the tumour to the cortex were measured as a function of tumour size and DMF treatment.

*Received 19 February 1996*
*Revised 24 September 1996*
*Accepted 30 September 1996*

*Correspondence to:* Y Boucher

*Present address: Department of Physiology, Temple School of Medicine, Broad and Ontario Streets Philadelphia, PA 19140, USA

### MATERIAL AND METHODS

### Animal protocol

#### Surgery and tumour implantation

Fisher rats (150–180 g) The animals were anaesthetized with a mixture of ketamine and xylazine (100 mg and 10 mg kg$^{-1}$). With a dentist's drill, two 3-mm holes were made through the bone on each side of the sagittal suture. The centre of the two holes was located 2 mm from the sagittal suture and 2 mm caudate to the coronal suture. In both openings the dura was removed. For pial surface tumours a piece of R3230AC or F98 tumour (1 × 1 × 1 mm) from a donor animal was introduced on the left side and a plastic cover slip was secured to the bone with cyanocrylate glue to close the opening. The other opening was also closed with a cover slip after introducing artificial CSF. Intraparenchymal tumours were also implanted following the preparation of a 3-mm opening in the bone at the same location as for pial surface tumours. Tumour slurry was prepared from pieces of R3230AC or F98 tumours and introduced into a Hamilton syringe linked to a 23-gauge needle. With a stereotaxic device the tip of the 23-gauge needle was placed 2 mm below the pial surface, and 20 μl of tumour slurry was injected. The opening was closed as previously described. To measure CSFP, 1-mm depressions occupying three-quarters of the bone thickness were drilled 1.5 mm from the sagittal suture and 0.5 mm caudate to the coronal suture.

As it was not possible to measure the microvascular pressure of the superficial microvessels of pial tumours through the 3-mm openings, an 8-mm opening was prepared over the two hemispheres with a dentist's drill. The dura was removed, and after introducing a piece of R3230AC the opening was closed with a plastic cover slip. The cover slip was perforated with four holes (diameter approximately 2 mm) and covered on one side with a thin transparent plastic wrap. Another cover slip was glued over the perforated cover slip.

#### Preparation of animals for IFP measurements

The animals were anaesthetized with chloral hydrate (300 mg kg$^{-1}$) and placed on a heating pad to maintain the body temperature between 36°C and 37°C. The left femoral artery was cannulated for measurement of arterial pressure and blood gases. The left femoral vein was cannulated for injection of saline, Evans blue or lissamine green. The animals were connected to an artificial ventilator following tracheotomy, and Pavalon (0.2 mg kg$^{-1}$) was given. All the IFP and MVP measurements were accepted as valid when the mean arterial pressure was greater than 70 mmHg and the $Pa\text{CO}_2$ was between 30 and 45 mmHg.

#### IFP and MVP measurements

IFP was measured with the micropipette technology described previously (Boucher et al, 1990; Boucher and Jain, 1992). In the R3230AC on the pial surface, IFP was measured in four different groups on day 6, 10, 14–15 and 17–20 after tumour implantation. The cover slip was removed, and artificial CSF (35–37°C) was superfused continuously on the tumour surface. In the smaller tumours on days 6 and 10, IFP was measured in the centre of the tumour at a distance of approximately 0.5–1.0 mm from the tumour surface. In the day 14–15 and 17–20 groups with the tumour thickness varying between 3.5 and 6.0 mm, only the IFP measurements obtained at a distance ≥ 0.5 mm from the tumour surface were used for data analysis. Following completion of the IFP measurements in the tumour, CSFP was measured through the 1-mm opening over the ventricle with a blunt 26-gauge needle connected to a pressure transducer. The thin layer of bone that remained in the 1-mm hole was easily perforated by carefully pushing the 26-gauge needle through the bone.

For measurement of MVP and IFP in the large windows, the first cover slip was removed and small holes were punctured through the transparent plastic wrap with a 30-gauge needle to have access to the tumour with micropipettes. Larger holes resulted in bulging of the tumour through the opening. MVP was measured in vessels with diameters between 40 and 100 μm. To verify that the tip of the micropipette was in the lumen of the tumour vessels, fast green (0.5%) was infused via the micropipette. MVP measurements were considered valid when the fast green disappeared rapidly with blood flow following the infusion. MVP was compared with the IFP measured at a distance ≥ 0.5 mm from the tumour surface.

Measurement in intraparenchymal tumours was done in R3230AC with tumour volumes between 50 and 175 mm$^3$ and in F98 gliomas with volumes between 10 and 70 mm$^3$. In both tumour types, the IFP was measured with micropipettes in the tumour and the surrounding brain.

**Table 1** Mean tumour volume and pressures in control and treated animals

| Tumour type and location | *n* | Tumour volume | Tumour IFP (mmHg) | Cortex IFP | CSFP |
|---|---|---|---|---|---|
| F98 parenchymal | | | | | |
| Small | 4 | 10.0 ± 2.5 | 9.0 ± 2.5$^{c,d}$ | 6.0 ± 1.0$^{a,c}$ | 5.5 ± 1.5$^{a,d}$ |
| Large | 4 | 29.0 ± 9.0 | 16.0 ± 6.0$^{a}$ | 18.0 ± 10.0$^{a,b}$ | 12.5 ± 5.5$^{a,b}$ |
| Large DMF treatment | 4 | 30.0 ± 26.0 | 4.5 ± 2.0$^{a,c,d}$ | 1.5 ± 1.0$^{b,c}$ | – 0.5 ± 1.5$^{b,c,d}$ |
| F98 pial surface | | | | | |
| Control | 4 | 31.0 ± 18.5 | 17.5 ± 8.0$^{c}$ | – | 7.0 ± 2.6$^{a,c}$ |
| DMF treatment | 4 | 39.0 ± 22.0 | 16.0 ± 4.0$^{c}$ | – | 1.5 ± 1.5$^{a,c}$ |
| R3230AC parenchymal | | | | | |
| Control | 4 | 109.0 ± 43.0 | 25.0 ± 10.0$^{a}$ | 21.0 ± 11.0$^{a}$ | 13.0 ± 5.0 |
| DMF treatment | 4 | 127.0 ± 50.0 | 6.0 ± 1.0$^{a}$ | 4.0 ± 1.0$^{a}$ | 7.0 ± 2.5 |
| R3230AC pial surface | | | | | |
| Control | 6 | 31.0 ± 21.0 | 19.5 ± 7.0$^{a,c}$ | – | 10.0 ± 4.5$^{a,c}$ |
| DMF treatment | 6 | 35.0 ± 25.0 | 8.0 ± 3.0$^{a,c}$ | – | 4.5 ± 1.5$^{a,c}$ |

$^{a,b}$ $P$<0.05 from top to bottom. $^{c,d}$ $P$<0.05 from left to right.

© Cancer Research Campaign 1997

**Figure 1(A)** Effect of tumour volume on the tumour IFP and CSFP in rats with R3230AC growing on the pial surface. Pressures represent the average of four to five animals for each time point (day 6, 10, 15 and 17–20) after tumour implantation. The increase in tumour pressure was linear with tumour volume ($y$=0.218 $x$ + 7.973, $r^2$ = 0.99). At small tumour volumes CSFP is independent of tumour size, at larger tumour size the increase in CSFP can be described by an exponential function ($y$=4.075 $(10)^{0.007x}$ $r^2$ = 0.969). □, Tumour IFP; ◆ CSFP. **(B)** Relationship between MVP and IFP in R3230AC located on the pial surface. The MVP was measured in superficial tumour vessels of 40–100 μm. As indicated by the line of equality, the IFP and MVP are closely related ($y$=4.5357 + 0.79682$x$ $r^2$ = 0.872, Spearman correlation $P$<.03)

### *Manipulation of tumour and cortex IFP, and CSFP*

Animals with F98 and R3230AC tumours implanted on the pial surface or in the parenchyma were treated with DMF. The animals were treated once daily with dexamethasone (3 mg $kg^{-1}$, i.p.), and for 3–4 days before the measurements. The last injection of dexamethasone was given 3–4 h before the IFP was measured. Mannitol (2.5 g $kg^{-1}$, at a rate of 0.25 ml $min^{-1}$ over a period of 10 min) was given first followed by furosemide (2 mg $kg^{-1}$) 15 min later. The pressure measurements were carried out under hypocapnia ($Paco_2$: 23–30 mmHg). The IFP was first measured with micropipettes 30–40 min after the start of mannitol infusion. CSFP was measured after the micropipette measurements. After the measurements, 2.0% Evans blue (0.2 ml 100 $g^{-1}$) or 2.0% lissamine green (0.5 ml 100$g^{-1}$) was injected via the femoral vein. The animals were killed 30 min following the injection of Evans blue and 5 min after lissamine green. The tumours were removed, and the tumour volume was estimated as $V = (\pi/6)\ a \times b^2$, where $a$ is the largest and $b$ is the smallest diameter of the tumour.

### *Statistical analysis*

The data are presented as the mean ± standard deviation. Significant differences between groups were determined by the Student's $t$-test ($P \leq 0.05$) or ANOVA. For an ANOVA with a $P \leq 0.05$, differences between groups were evaluated by the Bonferroni – Dunn multiple comparison test ($P \leq 0.016$). Data that were not normally distributed were analysed with the Wilcoxon signed-rank test.

### Human protocol

Pressures were measured in 11 neurosurgical patients undergoing resection for intracranial tumours. Before surgery, all patients had received dexamethasone (10 mg i.v.), mannitol (0.5 mg $kg^{-1}$ i.v.) and furosemide (20 mg i.v.) to reduce brain swelling during and after the opening of the intracranial cavity (Table 1). Hyperventilation was used to keep $Paco_2$ between 25 and 32 mmHg, and systemic arterial pressure was maintained at 120–140 mmHg. IFP was measured with the wick-in-needle technique (Boucher et al, 1991). The IFP in the tumours was measured following the opening of the intracranial cavity and removal of the dura. In five cases, IFP was also measured in the brain surrounding the tumour.

### RESULTS

### Measurements in rodents

To test if the opening of the intracranial cavity to the atmosphere would induce changes in intratumoral pressure and CSFP, both pressures were monitored following removal of the cover slip. The pressures remained stable after removal of the cover slip.

Figure 1A shows the change in tumour IFP and CSFP as a function of the tumour (R3230AC) volume on the pial surface. The tumour volumes varied between 1.0 and 230 $mm^3$, and to calculate the mean tumour volume and IFP the tumours were grouped in four different groups depending on the number of days after tumour implantation (Figure 1A). Tumour IFP increased with tumour size, while CSFP was not changed by the smaller tumour volumes but increased steeply at tumour volumes between 90 and 230 $mm^3$, which represents approximately 5–13% of the brain volume of a 200-g rat.

To assess the influence of $Paco_2$ on the tumour (R3230AC) IFP, the IFP was measured before and after modification of the rate and volume of ventilation in ten animals. In all the animals, there was a decrease in tumour IFP which varied between 17% and 50% at mean values of $Paco_2$, which decreased from 36 ± 2.5 to 24.5 ± 2.5 mmHg. The mean tumour IFP significantly decreased from 27 ± 15 to 18 ± 10 mmHg. The influence of $Paco_2$ on CSFP was also evaluated in five animals with tumours. With an average decrease in $Paco_2$ from 35.5 ± 1.5 to 23.5 ± 3.5 mmHg, CSFP significantly decreased from 31 ± 29 to 17 ± 15 mmHg.

© Cancer Research Campaign 1997

**Figure 2** Pressure profiles from F98 gliomas to the cortex in treated and non-treated animals with small and large tumours. The data points represent single IFP measurements and the dotted line indicates the tumour–cortex interface. The IFP increased at 2.0–2.5 mm from the pial surface in a rat with a tumour volume of 10 mm$^3$. In another animal with a tumour volume of 30 mm$^3$, the IFP increased at 1.0 mm or less from the pial surface. In contrast, in a rat with a large tumour (tumour volume 70 mm$^3$), treated with DMF and hypocapnia, the IFP increased at 2.0–2.5 mm from the pial surface. ◆, F98 small; △, F98 Large; □, F98 Large DMF

In pial surface tumours in large windows, the MVP of superficial vessels was compared with the IFP in the central regions of the tumours. The IFP was high and uniform in the tumour centre and generally dropped to lower values at 0.5 mm or less from the tumour surface. The IFP at ≥ 0.5 mm from the surface and the MVP were closely related (Figure 1B). The comparison between the MVP in superficial vessels and the central IFP assumes that the MVP of superficial and central vessels are similar (Boucher and Jain, 1992).

Treatment with DMF and hypocapnia induced a significant decrease in the IFP of R3230AC implanted on the pial surface. IFP was 19.5 ± 7.0 mmHg in the control group and 8.0 ± 3.0 mmHg in the treated group (Table 1). In F98 tumours on the pial surface, IFP was similar in the control and treated groups. In animals with F98 and R3230AC tumours, the CSFP was significantly decreased by DMF treatment and hypocapnia (Table 1).

The tumours implanted in the brain parenchyma were identified by the blue coloration resulting from the intravenous injection of lissamine green. In the F98 and R3230AC tumours implanted in the parenchyma, the tumour–cortex interface was located at 1.5–2.0 mm from the pial surface. For the pressure measurements, the micropipettes were introduced at depths of 3.5–4.0 mm from the pial surface; the IFP was measured for 20–30 s at intervals of 200–400 µm as the micropipette was retrieved to the pial surface. Depending on the fluid communication between the tumour and the micropipette, 3–8 IFP measurements were obtained thus permitting the characterization of IFP profiles from the tumour to the surrounding cortex. In animals with small F98 tumours (approximately 10 mm$^3$), the IFP (five out of seven pressure profiles) generally increased within a distance of 2.0–2.5 mm from the pial surface, which corresponds to the tumour–cortex interface or the peripheral regions of the tumours (Figure 2). In two pressure profiles, the IFP did not increase from the cortex to the tumour; the IFP in the tumour and in the cortex were similar. This type of IFP profile could be because of the location of the micropipette in the tumour periphery or in the surrounding cortex. In larger F98 tumours with volumes between 16 and 36 mm$^3$, all the IFP profiles were uniform except for the pressure increase at a distance of 1.0 mm or less from the pial surface (Figure 2). In contrast, in animals treated with DMF and with tumour volumes between 13 and 70 mm$^3$, IFP (eight out of nine pressure profiles) increased at 2.0–2.5 mm from the pial surface (Figure 2). The IFP in the tumour and cortex as well as the CSFP were significantly decreased by DMF treatment and hypocapnia (Table 1).

In R3230AC implanted in the parenchyma, the tumour volumes were larger than the F98 gliomas in both the control (52–150 mm$^3$) and treated (61–174 mm$^3$) groups. Similar to the larger F98 tumours, in animals with R3230AC, the IFP in the tumour and the cortex were similar (Table 1). The IFP increased at less than 0.5 mm from the pial surface. In animals treated with DMF and hypocapnia, the IFP (six out of eight pressure profiles) increased at 2.0–2.5 mm from the pial surface. Compared with the control group, DMF treatment and hypocapnia significantly reduced the IFP in the tumour and in the surrounding brain and induced a non-significantly decrease in CSFP (Table 1).

For all the intraparenchymal tumours, in order to calculate mean values of IFP, IFPs at > 2.0 mm from the pial surface were used to calculate the mean tumour IFP, whereas the mean IFPs in the cortex were determined from IFP measurements at less than 2.0 mm from the pial surface. This definition of the border between the tumour and the cortex was based on the location of the tumour–cortex interface at 1.5–2.0 mm from the pial surface and on the fact that, in animals with small F98 tumours or in animals treated with DMF, the IFP increased at 2.0–2.5 mm from the pial surface. The data were characterized by two types of analyses. In the type 1 analysis, all the IFP measurements in the tumour and in

**Table 2** Mean tumour volume and pressures in control and treated animals

| Tumour type and location | *n* | Tumour volume | Tumour IFP (mmHg) | Cortex IFP | CSFP |
|---|---|---|---|---|---|
| F98 parenchymal | | | | | |
| Small | 4 | 10.0 ± 2.5 | $11.0 \pm 1.5^{c,d}$ | $6.0 \pm 1.0^{a,c}$ | $5.5 \pm 1.5^{a,d}$ |
| Large | 4 | 29.0 ± 9.0 | $16.0 \pm 6.0^{a}$ | $18.0 \pm 10.0^{a,b}$ | $12.5 \pm 5.5^{a,b}$ |
| Large DMF treatment | 4 | 30.0 ± 26.0 | $5.5 \pm 2.5^{a,c,d}$ | $1.5 \pm 1.0^{b,c}$ | $-0.5 \pm 1.5^{b,d}$ |
| R3230AC | | | | | |
| Control | 4 | 109.0 ± 43.0 | $25.0 \pm 10.0^{a}$ | $21.0 \pm 11.0^{a}$ | 13.0 ± 5.0 |
| DMF treatment | 4 | 127.0 ± 50.0 | $7.0 \pm 1.0^{a,c}$ | $3.5 \pm 1.0^{a,c}$ | 7.0 ± 2.5 |

$^{a,b}P<0.05$ from top to bottom. $^{c,d}$ $P<0.05$ from left to right.

© Cancer Research Campaign 1997

**Table 3** Interstitial fluid pressure in human intracranial tumours

| Sex/age (years) | Tumour type | Previous treatment | Interstitial fluid pressure | |
|---|---|---|---|---|
| | | | Tumour | Brain surrounding tumour |
| M/30 | R-Glioblastoma | X | 3.0 | 3.5 |
| M/39 | R-Glioblastoma | X | 1.5 | 2.5 |
| F/51 | Glioblastoma | – | 0.5 | 0.5 |
| F/48 | Astrocytoma | – | 9.5 | 1.5 |
| F/70 | Meningioma | – | 1.5 | – |
| M/48 | R-Glioblastoma | S,X | 0.0 | – |
| F/36 | R-Glioblastoma | S,X | 1.0 | 3.0 |
| M/49 | Oligo/Astrocytoma | – | 2.5 | – |
| F/30 | R-Astrocytoma | S,C,X | 2.5 | – |
| M/36 | Ganglioglioma | – | 0.5 | – |
| F/62 | R-Astrocytoma | X,C,S | 2.0 | – |

Patients were treated with dexamethasone, mannitol and furosemide before surgery. R, recurrent; X, radiation; C, chemotherapy; S, surgery.

the cortex were included. The type II analysis included all the IFP profiles in the experimental groups with large F98 and R3230AC tumours as well as the IFP profiles that had an increasing IFP from the cortex to the tumour in animals treated with DMF and hypocapnia and animals with small F98 tumours. The exclusion of the IFP profiles that did not show a pressure increase from the cortex to the tumour was based on the possibility that the micropipettes could have been located in the periphery of the tumour or in the cortex surrounding the tumour. The type I and II analyses are given, respectively, in Table 1 and 2. In general, there was a good agreement between the results of the type I and II analyses. The main difference was found in R3230AC tumours treated with DMF. The type II analysis revealed that the IFP was significantly higher in the tumour than in the cortex (Table 2). With both types of analyses, there was a significant pressure difference between the tumour and the ventricle in animals with F98 tumours treated with DMF and hypocapnia, whereas, in animals with R3230AC tumours that were treated with DMF and hypocapnia, the CSFP and tumour IFP were similar (Table 1 and 2). There was no significant difference between the cortex IFP and the CSFP in treated and non-treated animals with F98 and R3230AC tumours. In general the cortex IFP was similar or higher than the CSFP, except in treated animals with large R3230AC tumours for which the CSFP was higher than the cortex IFP.

Following the intravenous injection of lissamine green both F98 and R3230AC became blue, thus demonstrating the absence of a blood–tumour barrier in these two tumour types. The treated and non-treated R3230AC and F98 tumours were also permeable to albumin as indicated by Evans blue.

### Measurements in patients

The IFP was measured in 11 patients with an astrocytoma, glioblastoma, meningioma or ganglioglioma (Table 3). The IFP in 10 of 11 tumours varied between 0.0 and 3.0 mmHg. In one astrocytoma the IFP was 9.5 mmHg. The mean IFP was 2.0 ± 2.5 mmHg. In five cases, the IFP measured in the brain surrounding the tumour varied between 0.5 and 3.5 mmHg. with a mean of 2.0 ± 1.0 mmHg (Table 3).

### DISCUSSION

One of the goals of the present study was to characterize the parameters that influence the IFP in intracranial tumours. Similar to peripheral tumours, the IFP in R3230AC on the pial surface increased with tumour size (Jain, 1987*a*; Boucher et al, 1990, 1991, 1995; Gutmann et al, 1992; Lee et al, 1992; Nathanson and Nelson, 1994). In R3230AC implanted on the pial surface, the MVP and IFP were similar (Figure 1B), thus suggesting that the increase in IFP with tumour size was due to the increasing MVP. In peripheral tumours as well as in tumours implanted on the pial surface, the high vascular permeability (Jain, 1987*b*; Yuan et al, 1994, 1995) and the absence of a functional lymphatic circulation (Taginawa et al, 1981) are probably responsible for the equilibration in oncotic and hydrostatic pressures between the microvascular and interstitial space (Jain, 1987*b*; Boucher and Jain, 1992). The equilibration of hydrostatic and oncotic pressures suggests that the MVP is the main driving force for interstitial hypertension and that modifications in arteriovenous pressure differences are responsible for the increase in IFP with tumour size. Increases in mean arterial pressure induced by angiotensin II as well as the artificial occlusion of the venous outflow of a tumour can significantly increase the tumour IFP (Wiig and Gadeholt, 1985; Zlotecki et al, 1993, 1995). Arteriovenous pressure modifications in the cerebral microcirculation could also contribute to the increase in tumour MVP and IFP, especially when the mass effect produced by the tumour induces a steep increase in CSFP (Figure 1A). It has been demonstrated that the cerebral venous pressure (CVP) increases in parallel with the CSFP and it is always kept slightly higher than the CSFP. Parallel modifications in CSFP and CVP can be induced by the inflation of intracranial balloons or by the modification of the $PaCO_2$ (Yada et al, 1973; Luce et al, 1982; Wiig and Reed, 1983). Thus, the increase in the CVP of the vessels draining the tumour could lead to a further increase in the tumour MVP and IFP.

Pressure gradients have been evaluated in the normal brain and after inducing lesions in the cortex. In two studies in normal brain, no pressure gradients were found between CSF and the grey or white matter (Poll et al, 1972; Wiig and Reed, 1983). Reulen and collaborators (Reulen and Kreysch, 1973; Reulen et al, 1977) measured pressures in the normal brain and in a cold lesion model of the cortex. Under normal conditions, they measured higher pressures in the CSF than in the white matter, and the lesion in the cortex induced higher pressures close to the lesion compared with the ventricles and distant sites in the white matter. Our results show that, in F98 gliomas with volumes of approximately 10 $mm^3$, there is a pressure gradient from the tumour to the cortex. At tumour volumes > 16 $mm^3$, which is approximately 1% of the brain volume of a rat, the IFP equilibrates between the tumour and the surrounding cortex (Figure 2 and Table 1). The decrease in pressure gradient from the tumour to the cortex is probably due to fluid filtration from tumour vessels. With increasing tumour size, the number of tumour vessels with high filtration rates increases thus surpassing the drainage capacity of the surrounding brain and thus inducing the pressure build-up. The pressure equilibration between the tumour and the surrounding tissue decreases the filtration of fluid towards an equilibrium that determines the extent of peritumoral oedema (Reulen et al, 1990). Pressure equilibration could be favoured by the absence of lymphatics in the cortex and the high resistance to bulk flow offered by the tortuous organization of the interstitial space of the grey matter (Fenstermacher,

© Cancer Research Campaign 1997

1984). In contrast, we have shown that the IFP was uniform throughout subcutaneous tumours and dropped steeply at the tumour–skin interface (Boucher et al, 1990).

### DMF treatment and hypocapnia

DMF treatment significantly decreased the IFP in the cortex and in F98 and R3230AC tumours localized in the parenchyma. Because of the larger pressure drop in the brain tissue, an IFP gradient was induced in both tumour types between the tumour and the surrounding cortex (Table 2). A pressure gradient was also induced by DMF treatment between F98 tumours and the ventricle, however no pressure gradient was found between R3230AC tumours and the ventricle. The larger tumour volume in R3230AC (mean volume 127 $mm^3$) compared with F98 tumours (mean volume 30.0 $mm^3$) could be responsible for the absence of pressure difference between the tumour and the ventricle. Because of the large size and thus close proximity to the ventricle, the R3230AC tumours could compress or obstruct the ventricle.

The presence of pressure gradients between the tumour and cortex or the tumour and ventricles in animals with small F98 tumours or following DMF treatment will favour the drainage of fluids and plasma proteins by bulk flow. In a cold-lesion model in the cortex, Reulen et al (1978) demonstrated that the clearance of water and albumin via the ventricles was significantly increased by experimentally inducing a pressure gradient between the cortex and the ventricles. The CSF is a significant pathway for the clearance of plasma proteins; 87% of the radioactive albumin cleared from the white matter can escape via the CSF system (Marmarou et al, 1994). The pressure gradients between the tumour and the cortex favours the drainage of peritumoral fluid via the subarachnoid space (Table 2). Arbit et al (1994) measured, in patients treated with dexamethasone alone (E Arbit, personal communication), a pressure gradient of 6.5 mmHg from the centre of brain tumours to the cortex. However, our measurements in patients with brain tumours did not reveal an IFP difference between the tumour and the surrounding brain, which could be due to the low IFP in the tumours and the limited number of patients in which the IFP was measured in both spaces.

The reduction in intraparenchymal and tumour IFP as well as in CSFP by DMF treatment and hypocapnia can be explained by the following mechanisms: (1) a reduction in vascular volume and pressure; (2) an increased resistance to the transvascular passage of water and serum proteins; (3) the osmotic or oncotic shifts of fluid from the interstitial space to blood; and 4) a reduction in CSF production. In the present study, the exact role of dexamethasone, mannitol and furosemide in the reduction of intracranial pressure or tumour IFP is impossible to define precisely as the three agents were used concomitantly. Furthermore, it is probable that each agent has more than one mechanism of action. Dexamethasone could reduce the tumour IFP and the intracranial pressure by decreasing the vascular permeability of tumour vessels or by reducing the vascular volume of the brain and brain tumours (Leenders et al, 1985; Reichman et al, 1986; Nakagawa et al, 1988; Neuwelt et al, 1993). Systemic infusions of mannitol can induce significant decreases in CSFP as well as in the water content of the normal brain and brain tumours (Bell et al, 1987; Ravussin et al, 1988; Hartwell and Sutton, 1993). The decrease in intracranial pressure induced by mannitol has been associated with osmotic shifts of water from the interstitial space to blood. Muizelaar et al (1983) have also proposed that the arteriolar vasoconstriction resulting from the systemic haemodilution induced by mannitol could reduce the cerebral blood volume and thus decrease CSFP. The diuretic furosemide can decrease intracranial pressure and potentiate the reduction in intracranial pressure with mannitol (Pollay et al, 1983; Albright et al, 1984). Reductions in intracranial pressure with furosemide may result from the inhibition of the reabsorption of water and sodium chloride by kidney tubules and/or the inhibition of CSF formation (McCarthy and Reed, 1974; Pollay et al, 1983). Reductions in CSF formation have also been demonstrated following treatment with mannitol and dexamethasone (Weiss and Nulsen, 1970; Donato et al., 1994).

DMF treatment and hypocapnia significantly reduced the intracranial pressure as well as the IFP in intraparenchymal tumours and R3230AC on the pial surface (Table 1). The reduction in intracranial pressure suggests that the decrease in the tumour MVP and IFP could be subsequent to a reduction in the CVP of the vessels draining the tumour. As mentioned previously, at low and high values of intracranial pressure the CVP is always kept slightly higher than the CSFP (Yada et al, 1973; Luce et al, 1982; Wiig and Reed, 1983). Wiig and Reed (1983) have shown that the CVP and CSFP change in parallel during the modulation of the $P\text{aco}_2$. In the present study in animals that were not treated with DMF, decreasing the $P\text{aco}_2$ from a mean of 36 mmHg to 24 mmHg significantly reduced the IFP in R3230AC on the pial surface and the CSFP by 33% and 45% respectively. Thus in animals that were treated with DMF and hypocapnia a portion of the decrease in tumour MVP and IFP was probably as a result of the decrease in CVP induced by the low $P\text{aco}_2$. Reductions in brain volume (e.g. decrease in peritumoral oedema) could also lead to a decrease in CVP and in tumour IFP, especially if the cerebral venous vessels are compressed by the expansion of the brain.

The reduction in the IFP of intraparenchymal tumours and R3230AC on the pial surface could also result from a decrease in water content induced by oncotic and osmotic gradients across tumour vessels. Hyperosmolar solutions have been shown to reduce the water content of brain tumours in patients and in a rodent model (Bell et al, 1987; Hansen et al, 1994). Hansen et al (1994) found with hypertonic sodium chloride a significant reduction in the water content of a brain glioma in the rat, which was impermeable to Evans blue, however hypertonic sodium chloride was not able to reduce the water content of a cold induced brain lesion permeable to Evans blue. The accumulation of Evans blue in treated and non-treated R3230AC and F98 tumours suggests that mannitol could equilibrate fairly rapidly across the microvascular wall, thus preventing the establishment of an effective osmotic gradient in those tumours. However, from the qualitative evaluation of the extravasation of Evans blue, it is impossible to determine if the oncotic gradient was increased by DMF treatment. As dexamethasone can reduce the accumulation of macromolecules in brain tumours (Reichman et al, 1986; Neuwelt et al, 1993), the oncotic gradient could be modified by DMF treatment.

### IFP in intracranial tumours in patients

In the present study, the mean IFP in brain tumours in patients was 2.0 mmHg, and Arbit et al (1994) recently reported a mean IFP of 7.2 mmHg by averaging the IFPs for meningiomas, glioblastomas and brain metastases. We speculated that the low IFP in brain tumours in patients was because of DMF treatment, hypocapnia and opening of the intracranial cavity as in normal human subjects CSFP can vary between 6 and 17.5 mmHg, with a mean around

© Cancer Research Campaign 1997

10 mmHg (Gilland et al, 1974; Corbett and Mehta, 1983), and untreated patients with brain tumours can have mean CSFPs varying between 24 and 33 mmHg depending on the studies (Kullberg and West, 1965; Miller and Leech, 1975; Alberti et al, 1978). Our experimental results confirm that the IFP in tumours on the pial surface and in the parenchyma is elevated, and that DMF treatment and hypocapnia can reduce significantly the IFP in intracranial tumours (Table 1). As it was impossible to measure the intratumoral pressure in a control group or before the opening of the intracranial cavity, it is not possible to conclude, in patients, that the low intratumoral IFP resulted exclusively from DMF treatment and hypocapnia. Fluid loss from the intracranial cavity can significantly reduce the CSFP (Lundberg and West, 1965; Gilland et al, 1974). It is also possible that, because of the lower vascular permeability of some brain tumours (Yuan et al, 1994), the IFP could be low or similar to the CSFP, especially in tumours not influenced by growth in a confined space. However, even in a tumour with a low vascular permeability, the pressure could be elevated as a result of equilibration with the intracranial pressure.

In summary, the results demonstrate that, similar to some peripheral tumours, the IFP of R3230AC on the pial surface increased with tumour size and the tumour MVP was similar to the IFP. In contrast to subcutaneous tumours, the IFP did not decrease in the periphery of the larger tumours in the cortex; the pressure in the surrounding cortex and in the tumour were equal. Treatment with DMF significantly decreased the IFP of brain tumours and the intracranial pressure, and induced pressure gradients between the tumours and the cortex. The low IFP values in intracranial tumours in patients are due in part to DMF treatment and hypocapnia.

### ACKNOWLEDGEMENTS

We thank Drs Larry Baxter, Claus A Kristensen and Fan Yuan for their helpful comments on the present manuscript. The F98 tumour was a kind gift of Dr Rolf F Barth. This study was supported by an NCI Outstanding Investigator Award (R35 CA56591) to RKJ.

### REFERENCES

Alberti E, Hartmann A, Schutz HJ and Schreckenberger F (1978) The effect of large doses of dexamethasone on the cerebrospinal fluid pressure in patients with supratentorial tumors. *J Neurol* **217**: 173–181

Albright AL, Latchaw, RE and Robinson AG (1984) Intracranial and systemic effects of osmotic and oncotic therapy in experimental cerebral edema. *J Neurosurg* **60**: 841–849.

Arbit E, Lee J and Diresta, G (1994) Interstitial hypertension in human brain tumors: possible role in peritumoral edema formation. In *Intracranial Pressure IX* Nagai H and Kayima K (eds), pp. 609–614. Springer: Tokyo

Baxter LT and Jain RK (1989) Transport of fluid and macromolecules in tumors. I Role of interstitial pressure and convection. *Microvascular Res* **37**: 77–104

Bell BA, Kean DM, MacDonald HL, Barnett G H, Douglas RHB, Smith MA, Mcghee CNJ, Miller JD, Tocher JL and Best JJK (1987) Brain water measured by magnetic resonance imaging. Correlation with direct estimation and changes after mannitol and dexamethasone. *Lancet* **1**: 66–69

Boucher Y and Jain RK (1992) Microvascular pressure is the principal driving force for interstitial hypertension in solid tumors: implications for vascular collapse. *Cancer Res* **52**: 5110–5114

Boucher Y, Baxter L and Jain RK (1990) Interstitial pressure gradients in tissue-isolated and subcutaneous tumors: implications for therapy. *Cancer Res* **50**: 4478–4484

Boucher Y, Kirkwood JM, Opacic D, Desantis M and Jain RK (1991) Interstitial hypertension in superficial metastatic melanomas in patients. *Cancer Res* **51**: 6691–6694

Boucher Y, Lee I and Jain RK (1995) Lack of general correlation between interstitial fluid pressure and oxygen partial pressure in solid tumors. *Microvasc Res* **50**: 175–182

Corbett JJ and Mehta MP (1983) Cerebrospinal fluid pressure in normal obese subjects and patients with pseudotumor cerebri. *Neurol* **33**: 1386–1388

Curti BD, Urba WJ, Alvord WG, Janik JE, Smith JW, Madara K and Longo DL (1993) Interstitial pressure of subcutaneous nodules in melanoma and lymphoma patients: changes during treatment. *Cancer Res* **53**: 2204–2207

DiResta GR, Lee J, Larson SM and Arbit E (1993) Characterization of neuroblastoma xenograft in rat flank. I Growth interstitial fluid pressure and interstitial fluid velocity profiles. *Microvascular Res* **46**: 158–177

Donato T, Shapira Y, Artru A and Powers K (1994) Effect of mannitol on cerebrospinal fluid dynamics and brain tissue edema. *Anesth Analg* **78**: 58–66

Fenstermacher J (1984) Volume regulation of the central nervous system. In *Edema*. Staub NC and Taylor AE (eds), pp. 383–404 Raven Press: New York

Gilland O, Tourtellotte WW, O'Tauma L and Henderson WG (1974) Normal cerebrospinal fluid pressure. *J Neurosurg* **40**: 587–593

Gutmann R, Leunig M, Feyh J, Goetz AE, Messmer K, Kastenbauer E and Jain R K (1992) Interstitial hypertension in head and neck tumors in patients: correlation with tumor size. *Cancer Res* **52**: 1993–1995

Hansen TD, Warner DS, Traynelis VC and Todd MM (1994) Plasma osmolality and brain water content in a rat glioma model. *Neurosurgery* **34**: 505–511

Hartwell RC and Sutton LN (1993) Mannitol, intracranial pressure and vasogenic edema. *Neurosurgery* **32**: 444–450

Jain RK (1987*a*) Transport of molecules in the tumor interstitium: a review. *Cancer Res* **47**: 3038–3050

Jain RK (1987*b*) Transport of molecules across tumor vasculature. *Cancer Met Rev* **6**: 559–594

Jain RK and Baxter LT (1988) Mechanisms of heterogeneous distribution of monoclonal antibodies and other macromolecules in tumors: significance of elevated interstitial pressure. *Cancer Res* **48**: 7022–7032

Kullberg G and West KA (1965) Influence of corticosteroids on the ventricular fluid pressure. *Acta Neurol Scand Suppl* **13**: 445–452

Lee I, Boucher Y and Jain RK (1992) Nicotinamide can lower tumor interstitial fluid pressure: mechanistic and therapeutic implications. *Cancer Res* **52**: 3237–3240

Leenders KL, Beaney RP, Brooks DJ, Lammertsma AA, Heather JD and McKenzie C G (1985) Dexamethasone treatment of brain tumor patients: effects on regional cerebral blood flow, blood volume, and oxygen utilization. *Neurology* **35**: 1610–1616

Less JR, Posner MC, Boucher Y, Borochowitz D, Wolmark N and Jain RK (1992) Interstitial hypertension in human breast and colorectal tumors. *Cancer Res* **52**: 6371–6374

Luce JM, Huseby JS, Kirk W and Butler J (1982) A starling resistor regulates cerebral venous outflow in dogs. *J Appl Physiol* **53**: 1496–1503

Lundberg N and West KA (1965) Leakage as a source of error in measurement of the cerebrospinal fluid pressure by lumbar puncture. *Acta Neurol Scand* **41**: 115–121

Marmarou A, Hochwald G, Nakamura T, Tanaka K, Weaver J and Dunbar J (1994) Brain edema resolution by CSF pathways and brain vasculature in cats. *Am J Physiol* **267**: H514–H520

McCarthy KD and Reed DJ (1974) The effect of acetazolamide and furosemide on cerebrospinal fluid production and choroid plexus carbonic anhydrase activity. *J Pharmacol Exper Therapeutics* **189**: 194–201

Miller JD and Leech P (1975) Effects of mannitol and steroid therapy on intracranial volume–pressure relationships in patients. *J Neurosurg* **42**: 274–281

Muizelaar JP, Wei EP, Kontos HA and Becker DP (1983) Mannitol causes compensatory vasoconstriction and vasodilatation in response to blood viscosity changes. *J Neurosurg* **59**: 822–828

Nakagawa H, Groothuis DR, Owens ES, Patlak C, Pettigrew KD and Blasberg R R (1988) Dexamethasone effects on vascular volume and tissue hematocrit in experimental RG-2 gliomas and adjacent brain. *J Neuro-Oncol* **6**: 157–168

Nathanson SD and Nelson L (1994) Interstitial fluid pressure in breast cancer, benign breast conditions, and breast parenchyma. *Ann Surg Oncol* **1**: 333–338

Neuwelt EA, Barnett PA, Ramsey FL, Hellstrom MD, Hellstrom KE and McCormick C I (1993) Dexamethasone decreases the delivery of tumor-specific monoclonal antibody to both intracerebral and subcutaneous tumor xenografts. *Neurosurgery* **33**: 478–484

Poll W, Brock M, Markakis E, Winkelmuller W and Dietz H (1972) Brain tissue pressure. In *Intracranial Pressure,* Brock M and Dietz H (eds), pp. 185–187 Springer-Verlag: New York

Pollay M, Fullenwider C, Roberts A and Stevens A (1983) Effect of mannitol and furosemide on blood–brain osmotic gradient and intracranial pressure. *J Neurosurg* **59**: 945–950

© Cancer Research Campaign 1997 

Ravussin P, Abou- Madi M, Archer D, Chiolero R, Freeman J, Trop D and De Tribolet N (1988) Changes in CSF pressure after mannitol in patients with and without elevated CSF pressure. *J Neurosurg* **69:** 869–876

Reichman HR, Farrell CL and Del Maestro RF (1986) Effects of steroids and non steroid anti-inflammatory agents on vascular permeability in a rat glioma model. *J Neurosurg* **65:** 233–237

Reulen HJ and Kreysch HG (1973) Measurement of brain tissue pressure in cold induced cerebral oedema. *Acta Neurochir* **29:** 29–40

Reulen HJ, Graham R, Spatz M and Klatzo I (1977) Role of pressure gradients in bulk flow in dynamics of vasogenic brain edema. *J Neurosurg* **46:** 24–35

Reulen HJ, Tsuyumu M, Tack A, Fenske AR and Prioleau GR (1978) Clearance of edema fluid into cerebrospinal fluid. *J Neurosurg* **48:** 754–764

Reulen HJ, Huler P, Ito U and Groger U (1990) Peritumoral brain edema: a keynote address. *Adv Neurol* **52:** 307–315

Roh HD, Boucher Y, Kalnicki S, Buchsbaum R, Bloomer WD and Jain RK (1991) Interstitial hypertension in carcinoma of uterine cervix in patients: possible correlation with tumor oxygenation and radiation response. *Cancer Res* **51:** 6695–6698

Stohrer M, Boucher Y, Stangassinger M and Jain RK (1995) Oncotic pressures in human tumor xenografts. *Proc Amer Assoc Cancer Res* **36:** 311

Sundbarg G, Nordstrom CH, Messeter K and Sodestrom S (1987) A comparison of intraparenchymatous pressure recording in clinical practice. *J Neurosurg* **67:** 841–845

Taginawa N, Kanazawa T, Satomura K, Hikasa Y, Hashida M, Muranishi S and Sezaki H (1981) Experimental study on lymphatic vascular changes in the development of cancer. *Lymphology* **14:** 149–154

Tufto I and Rofstad EK (1995) Interstitial fluid pressure in human melanoma xenografts. *Acta Oncol* **34:** 361–365

Weiss MH and Nulsen FE (1970) The effect of glucocorticoids on CSF flow in dogs. *J Neurosurg* **32:** 452–458

Wiig H and Reed RK (1983) Rat brain interstitial fluid pressure measured with micropipettes. *Am J Physiol* **244:** H239–H246

Wiig H and Gadeholt G (1985) Interstitial fluid pressure and hemodynamics in a sarcoma implanted in the rat tail. *Microvasc Res* **29:** 176–189

Yada K, Nagakawa Y and Tsuro M (1973) Circulatory disturbance of the venous system during experimental intracranial hypertension. *J Neurosurg* **39:** 723–729

Yuan F, Salehi HA, Boucher Y, Vasthare US, Tuma F and Jain RK (1994) Vascular permeability of gliomas and mammary carcinomas transplanted in rat and mouse cranial windows. *Cancer Res* **54:** 4564–4568

Yuan F, Dellian M, Fukumura D, Leunig M, Berk DA, Torchilin VP and Jain RK (1995) Vascular permeability in a human tumor xenograft: molecular size dependence and cut-off size. *Cancer Res* **55:** 3752–3756

Zlotecki RA, Boucher Y, Lee I, Baxter LT and Jain RK (1993) Effect of angiotensin II induced hypertension on tumor blood flow and interstitial fluid pressure. *Cancer Res* **53:** 2466–2468

Zlotecki RA, Baxter LT, Boucher Y and Jain RK (1995) Pharmacologic modification of tumor blood flow and interstitial fluid pressure in a human tumor xenograft: network analysis and mechanistic interpretation. *Microvasc Res* **50:** 429–443

Znati CA, Rosenstein M, Boucher Y, Epperly MW, Bloomer WD and Jain RK (1996) Effect of radiation on interstitial fluid pressure and oxygenation in a human tumor xenograft. *Cancer Res* **56:** 964–968

*© Cancer Research Campaign 1997*